# LMX1B rs10733682 Polymorphism Interacts with Macronutrients, Dietary Patterns on the Risk of Obesity in Han Chinese Girls

**DOI:** 10.3390/nu12051227

**Published:** 2020-04-26

**Authors:** Qi Zhu, Kun Xue, Hong Wei Guo, Yu Huan Yang

**Affiliations:** 1 Department of Medicine, Xinglin College, Nantong University, Nantong 226008, China; zgjsntzq@126.com; 2 Department of Nutrition, School of Public Health, Fudan University, Shanghai 200032, China; xuekun@shmu.edu.cn; 3 Department of Preventive Medicine, School of Public Health, Nantong University, Nantong 226019, China

**Keywords:** LMX1B rs10733682 polymorphism, macronutrients, dietary patterns, childhood obesity, lipid profile, interaction analysis

## Abstract

Previous studies have found that LMX1B rs10733682 polymorphism is associated with Body Mass Index (BMI) in European and American Indian adults. In this study, the association of rs10733682 polymorphism with obesity-related indicators, and its interaction with macronutrients and dietary patterns (DPs) were explored in Chinese children (*n* = 798). The rs10733682 polymorphism was genotyped by improved Multiple Ligase Detection Reaction (iMLDR). Four DPs were identified by factor analysis. The AA genotype had a higher incidence of overweight/obesity than GG+GA genotypes (*P* = 0.010) in girls (*n* = 398), but no difference in boys. The AA genotype in girls could interact with intake of energy, fat and carbohydrate, causing an increased triglyceride (TG), (*P* = 0.021, 0.003, 0.002, respectively), and also could interact with energy from protein, causing an elevated BMI (*P* = 0.023) and waist (*P* = 0.019). Girls inclining to the HED (high-energy density)-DP were associated with increased TG (*P* = 0.033), and girls inclining to the VEF (vegetables, eggs, and fishes based)-DP were associated with decreased total cholesterol (TC, *P* = 0.045) and decreased low density lipoprotein cholesterin (LDL, *P* = 0.016). The findings indicated that the AA genotype of rs10733682 and the HED-DP are potential risk factors of obesity in Chinese girls.

## 1. Introduction

The epidemic of overweightness and obesity has been observed from childhood, through adolescence to adulthood [1]. Childhood obesity is often accompanied with a risk of cardiovascular disease and metabolic syndrome, and associated with psychosocial problems, hypertension, dyslipidemia, type 2 diabetes, and even premature mortality in adulthood [2,3,4], as well as with an increased economic burden on individuals and society [5]. The incidence of childhood overweight and obesity in China continued to increase dramatically from 1991 to 2015 [6,7], which has become an urgent public health concern and requires unremitting research efforts to illuminate its causes and risk factors.

From the perspective of traditional nutrition, environmental risk factors such as excessive energy intake, unbalanced dietary patterns, and physical inactivity are associated with the development of obesity. Also, medical conditions, including Leptin imbalance, hypothyroidism, and medication, including steroid medications for mental health, may increase weight. With the development of modern molecular nutrition, it has been gradually perceived that obesity arises from the interaction between genetic profile and external environmental risk factors [8], as well as epigenetic effects on obesity in an intrauterine environment [9]. Sex, race, and even individuals have different susceptibility to these factors, which provide new insights into the variation of obesity susceptibility [10]. Single nucleotide polymorphism (SNP) is the most common of the human heritable variation, accounting for more than 90% of all known polymorphisms. To date, several of the most studied SNPs, including FTO (fat mass and obesity-related) gene, MC4R (melanocortin receptor) gene, and others [11,12,13], have made researchers increasingly aware of the impact of SNP on the development of obesity. For example, a meta-analysis (including studies published by the end of September 2014, total 213,173 adults, ages range 31–75 years) indicated that the FTO risk allele is associated with energy intake in adults, and also showed the role of FTO in changing the proportions of fat and protein energy expenditure [14]. As we know, the synergetic risk of obesity is likely to be linked to the accumulation or interaction of multiple SNPs, each of which contributes to a fraction of the total risk. Therefore, more susceptibility gene polymorphisms are being mined to deepen understanding of the causes of obesity [15].

The LMX1B gene located on autosomal 9q34.1 belongs to the transcription factor family and is mainly involved in protein binding. The LMX1B gene is widely expressed in vertebrate embryos and plays multiple functions in the development of kidney, limbs and central nervous system [16,17,18]. A largest (based on a total of 322,154 European descent adults) genome-wide association study (GWAS) and Metabochip meta-analysis to date identified 56 new genetic loci significantly associated with BMI, among which LMX1B rs10733682 is one of them [19]. A latest study also found that the LMX1B rs10733682 is associated with BMI in Pima Indian adults [20]. Another LMX1B polymorphism loci rs3829849 was found to be associated with obesity in Central Mexican children [21]. Furthermore, genetic variants associated with BMI may also influence eating behavior, and it has been shown that the LMX1B rs10733682 is nominally associated with satiety responsiveness [22].

However, whether there is a significant association between the LMX1B rs10733682 polymorphism and obesity in Chinese children remains to be verified. To our knowledge, there have been no studies on the relationship between the incidence of obesity, obesity-related indicators and the LMX1B rs10733682 polymorphism in Chinese children. The purpose of this study was to reveal the association between the LMX1B rs10733682 polymorphism and obesity, as well as to explore the interaction of the LMX1B rs10733682 polymorphism with macronutrients intake, dietary patterns and physical activity levels on obesity risk of Chinese children. It is hoped that this will provide clues for a systematic study on the mechanism of obesity in children and the establishment of personalized preventive and control measures in the future.

## 2. Materials and Methods

### 2.1. Study Population

Data were obtained from the baseline investigation of nutrition-based comprehensive intervention study on childhood obesity in China (NISCOC, Chinese clinical trial registry (primary registry in the WHO registry network) identifier: ChiCTR-TRC-00000402). It was approved by the ethics review committee of the institute of Nutrition and Food Safety, Chinese Center for Disease Control and Prevention (Ethics No.: 20081201). The sampling area was divided into three layers: urban, suburban, and rural in Shanghai. Two schools were randomly selected in each layer, and then two classes were randomly selected from grade 1 ~ 4. Lastly, about 20 pupils (sick or medicated not included) were randomly drawn from the classes as the participants. All participants including their parents or legal guardians had been informed of the purpose and procedures of the study and had signed informed consents.

### 2.2. Anthropometric Assessments

Height and fasting weight was measured using electronic scale (GMCS-I; Yishen, Shanghai, China; the accuracy is greater than ± 0.1%), while the participants were minimally clothed and barefoot. Then BMI was calculated as weight/height squared (kg/m^2^). The participants were classified as obesity, overweight, and normal weight in accordance with the “Chinese school-age children and adolescents overweight and obesity screening BMI classification criteria” proposed by Working Group on Obesity in China (WGOC) [23]. Waist circumference (WC) was measured at the end of a normal exhalation by an unstretched tape (accuracy of 0.1 cm) at the umbilical level, without any pressure to the body surface. If the participant was obese, the measurement should be made around the widest position. The waist-to-height ratio (WHtR) was calculated, results greater than 0.47 (for boys) and 0.45 (for girls) were classified as central obesity (also known as abdominal obesity) [24]. Blood pressure was measured after a rest of at least 10-min by a mercury sphygmomanometer. All of the above measurements were repeated twice and then averaged. Basal metabolic rate (BMR) was calculated according to the prediction equation recommended by report of a joint FAO/WHO/UNU expert consultation [25].

### 2.3. Dietary and Physical Activity Assessments

A standardized format of food record form with food size labels was used to record the type and amount of the foods participants ate at home and elsewhere for three consecutive days (including 2 school days and 1 weekend). Afterwards, the daily energy and nutrient intake of each participant was calculated through a self-developed SY Nutrients Analysis and Formulation software [26]. All foods were converted into 21 food groups to extract dietary patterns.

Participants’ physical activities, including dynamic duration (walking, cycling, exercise, etc.), static duration (doing homework, playing on the phone or computer, watching TV, etc.), and sleep duration, were reviewed using a seven-day retrospective physical activity questionnaire (translated from “International Physical Activity Questionnaire-short”) [27]. Subsequently, according to Ainsworth [28], the dynamic duration was converted to MET-h/wk (metabolic equivalent hours per week).

### 2.4. Laboratory Assays

After overnight fasting, blood samples were drawn early in the morning. Then serum triglycerides (TG), total cholesterol (TC), high density lipoprotein cholesterol (HDL), low density lipoprotein cholesterol (LDL), and fasting blood glucose (FBG) were assayed by standard protocols (Enzyme Kit: Fenghui Medical Technology Company, Shanghai, China; LX-20 automatic biochemical analyzer: Beckman Clouter, CA, USA).

### 2.5. Genotyping

DNA was extracted from whole blood by the phenol-chloroform method. The LMX1B rs10733682 was genotyped by iMLDR technique at GENESKY Biotechnology Company (Shanghai, China). PCR used the following primers: forward 5′-CAGCCCCAGTCTGCTCAACTCT-3′; reverse 5′-ACGGGAGCAAAACCACAGAACA-3′ (Sangon Biotech, Shanghai, China). PCR reactions were performed in a final volume of 20 μL, containing 1μL extracted DNA, 1 μL primers, 1* GC-I buffer (Takara, Shanghai, China), 3.0 mM Mg^2+^, 0.3 mM dNTP (Generay Biotech, Shanghai, China), and 1 U HotStarTaq polymerase (Qiagen, Dusseldorf, NW, Germany) with the following conditions in a DNA thermocycler: DNA templates were denatured at 95 °C for 2 min; amplification consisted of 11 cycles at 94 °C for 20 s, 65 °C for 40 s (−0.5 °C/cycle), 72 °C for 1.5 min, and 24 cycles at 94 °C for 20 s, 59 °C for 30 s, 72 °C for 1.5 min, with a final extension at 72 °C for 2 min. Amplified DNA(20 μL) was digested with a 5 U SAP enzyme (Promega, Madison, WI, USA) and 2 U Exonuclease I enzyme (Epicentre, US) at 37 °C for 1 h, and then inactivated at 75 °C for 15 min. Ligase chain reaction (LCR) used the following primers: RG: 5′-TCTCTCGGGTCAATTCGTCCTTCCTAGGGCTTGACCTCAGAGTTAAATGC-3′; RA: 5′-TGTTCGTGGGCCGGATTAGTCCTAGGGCTTGACCTCAGAGTTAAACGT-3′; RP: 5′-AGGGTTGTGAGCACCTGAACA-3′ (Sangon Biotech, Shanghai, China). LCR reactions system contained 1 μL 10* ligation buffer, 0.25 μL high temperature ligase (Thermo Fisher Scientific, Waltham, MA, USA), 0.4 μL 5′primers (1 μM), 0.4 μL 3′primers (2 μM), 2μL purified product of PCR, and 6 μL ddH2O with the following conditions: 38 cycles at 94 °C for 1 min and 56 °C for 4 min. Dilute ligation product (0.5 μL) was blended with 0.5 μL Liz500 Size Standard, 9 μL Hi-Di (Applied Biosystems, Foster City, CA,, USA), and denatured at 95 °C for 5 min. The sequencing process was performed using the ABI3730XL automated sequencer (Applied Biosystems, Foster City, CA, USA). The original data collected from the sequencer were analyzed with GeneMapper 4.1 (Applied Biosystems, Foster City, CA, USA).

### 2.6. Statistical Analyses

This study used the Statistical Package for the Social Science (SPSS version 22.0, IBM, Armonk City, NY, USA) for statistical analysis. The independent *t*-test was used to compare the blood pressure and lipid profiles indicators between the obese and normal weight groups. Pearson’s χ^2^ test was used to verify the Hardy-Weinberg equilibrium. Logistic regression was used to compare the incidence of overweight/obesity and central obesity between genotypes, and odds ratio (OR) and 95% confidence intervals (95% CI) were calculated. Dietary patterns were extracted by principal component factor analysis with varimax rotation and based on 21 food groups, and adjusted for total energy intake by way of residual regression. Dietary patterns were retained according to criteria including a scree plot and an eigenvalue greater than 1.5. Afterwards, all obesity-related indicators were quartile from lowest (Q1) to highest (Q4) according to the macronutrients intake, dietary patterns factor score, BMR and physical activity duration, respectively. Analysis of variance (ANOVA) and LSD post-hoc test were used to compare the quantitative variables, and the confounders were adjusted for by analysis of covariance (ANCOVA). Multiple linear regression was used to analyze the interactions between the LMX1B rs10733682 polymorphism and macronutrients intake, dietary patterns, and physical activity duration, respectively. *P* value < 0.05 was considered significant, and *P* values between 0.05 and 0.1 were considered as marginally significant for interaction analysis.

## 3. Results

A total of 798 participants were included in the study after excluding null and invalid samples, of which there were 400 (50 %) boys and 398 (50%) girls. The age span ranges from 7 to 12 years, with an average age of 9.2 ± 0.9. It was found that the incidence rate of general overweight/obesity and central obesity was 21% (boys 26% and girls 16%) and 16% (boys 19% and girls 13%), respectively.

### 3.1. Association between Obesity-Related Indicators and LMX1B rs10733682 SNP

In our study, the frequency of the A allele was 76% (this is 75% in American Indian and 43% in European [20]) and the frequency of G allele (MAF) was 24% in Han Chinese children. There were three genotypes: AA (58%), AG (37%), and GG (5%), which did not deviate from Hardy-Weinberg equilibrium expectations (*P* = 0.853). 

Girls with the AA genotype showed a significantly higher overweight/obesity incidence (20%) than those with the GA+GG genotypes (10%) before (*P* = 0.011; OR 2.192; 95% CI 1.196–4.018) and after (*P* = 0.010; OR 2.196; 95% CI 1.195–4.034) adjusting for potential confounders (age, total energy intake, physical activity duration). Girls with the AA genotype also had a higher central obesity incidence (16%) compared to the GG+GA genotypes (10%) before (*P* = 0.047; OR 1.77; 95% CI 0.93–3.34) and after (*P* = 0.045; OR 1.78; 95% CI 0.94–3.35) adjusting for potential confounders. Girls with the AA genotype also exhibited a higher body weight, BMI, WC, WHtR, and BMR before and after adjusting for potential confounders (Table 1). However, there were no significant differences in boys according to the genotype (not shown). Because of that, boys were not included in the following analysis.

Compared to the normal weight group, both of the general overweight/obesity and central obesity girls showed significantly higher SP, DP, TG, and LDL, as well as lower HDL levels. The general overweight/obesity group also exhibited a significantly higher FBG level than normal weight group (Table 2). 

### 3.2. Association and Interaction between Obesity-Related Indicators, Macronutrients Intake, and LMX1B rs10733682 SNP

Girls who had an increased intake of total energy, energy from fat, and fat intake were associated with higher TG level, but an increased intake of energy from protein were associated with lower TG level, both before and after adjustment for confounders (age and physical activity duration). Additionally, girls who had an increased intake of carbohydrate and dietary fiber were associated with lower TC level, both before and after adjustment for confounders (Table 3). There were no significant differences of other obesity-related indices between the quartile groups of macronutrients intake for girls (not shown).

**Figure 1:**
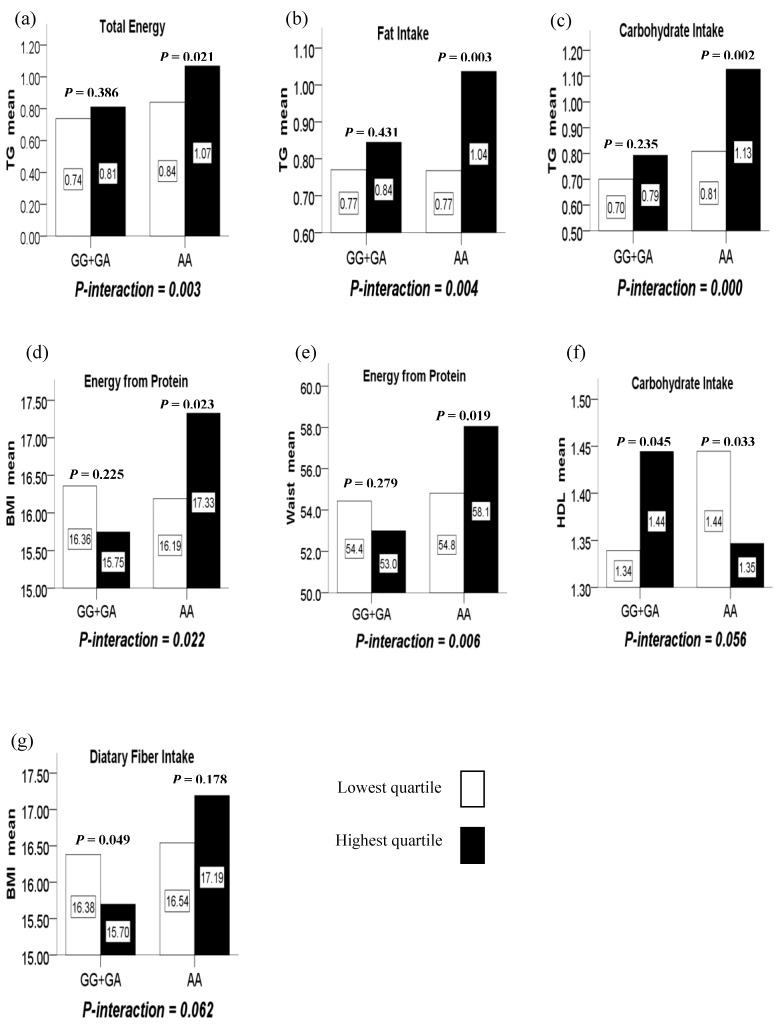
(**a**) The interaction between the LMX1B rs10733682 polymorphism and total energy intake on TG; (**b**) the interaction between the LMX1B rs10733682 polymorphism and fat intake on TG; (**c**) the interaction between the LMX1B rs10733682 polymorphism and carbohydrate intake on TG; (**d**) the interaction between the LMX1B rs10733682 polymorphism and energy intake from protein on BMI; (**e**) the interaction between the LMX1B rs10733682 polymorphism and energy intake from protein on WC; (**f**) the marginal interaction between the LMX1B rs10733682 polymorphism and carbohydrate intake on HDL; (**g**) the marginal interaction between the LMX1B rs10733682 polymorphism and dietary fiber intake on BMI.

There were no significant differences in the macronutrients intake between girls with different genotypes (not shown), but some following interactions were observed through further analysis:

(1) There were significant interactions between intake of total energy, fat, carbohydrate and the LMX1B rs10733682 polymorphism in terms of TG (*P*-interaction = 0.003, 0.004, and 0.000, respectively). In such a way, the highest quartile of total energy, fat and carbohydrate intake compared to the lowest quartile intake, increased TG level with the AA genotype (*P* = 0.021, 0.003, and 0.002, respectively) while having no difference for the GG+GA genotypes (*P* = 0.386, 0.431, and 0.235, respectively). (Figure 1a–c)

(2) There were significant interactions between the LMX1B rs10733682 polymorphism and energy from protein intake in terms of BMI and WC (*P*-interaction = 0.022 and 0.006). In such a way, the highest quartile of the energy from protein intake compared to the lowest quartile, increased BMI and WC level with the AA genotype (*P* = 0.023 and 0.019) while having no difference for GG+GA genotypes (*P* = 0.225 and 0.279). (Figure 1d,e)

(3) There were marginally significant interactions between the LMX1B rs10733682 polymorphism and carbohydrate intake in terms of HDL (*P*-interaction = 0.056). In such a way, the highest quartile of the carbohydrate intake compared to the lowest quartile, reduced HDL level with the AA genotype (*P* = 0.033), but increased with the GG+GA genotypes (*P* = 0.045). (Figure 1f)

(4) There were marginally significant interactions between the LMX1B rs10733682 polymorphism and dietary fiber intake in terms of BMI (*P*-interaction = 0.062). In such a way, the highest quartile of the dietary fiber intake compared to the lowest quartile, reduced BMI level with the GG+GA genotypes (*P* = 0.049) while having no difference for the AA genotype (*P* = 0.178). (Figure 1g).

### 3.3. Association and Interaction between Obesity-Related Indicators, Dietary Patterns, Physical Activity Duration, and LMX1B rs10733682 SNP

Based on the dietary data, four major dietary patterns of girls were identified: (1) high-energy density dietary patterns (HED-DP); (2) nuts, pork, and wheat-based dietary patters (NPW-DP); (3) vegetables, eggs, and fishes based dietary patterns (VEF-DP); (4) dairy, fruits, eggs-based dietary patterns (DFE-DP). The HED-DP was characterized by a higher consumption of various high-energy density foods, such as pastries, meats, and sweets. The NPW-DP was characterized by a higher consumption of nuts, pork, and wheaten. The VEF-DP was characterized by a higher intake of vegetables, eggs and fishes. The DFE-DP was higher in dairy, fruits, eggs, and sweets. (Table 4)

The characteristics of girls across quartile categories of the dietary pattern factor scores were shown in Table 5. Girls in the highest quartile (Q4) of HED-DP had a significantly higher total energy intake and energy intake from carbohydrate, but lower energy from protein than the lowest quartile (Q1). Girls in the highest quartile of NPW-DP had a significantly higher energy intake from fat, but lower energy from carbohydrate than the lowest quartile. Girls in the highest quartile of VEF-DP had a significantly higher energy intake from protein, fat, but lower energy from carbohydrate than the lowest quartile. Girls inclining to HED-DP were associated with higher TG level, and inclining to the VEF-DP was associated with lower levels of TCH and LDL. There were no significant differences between other obesity-related indices (not shown).

**Figure 2:**
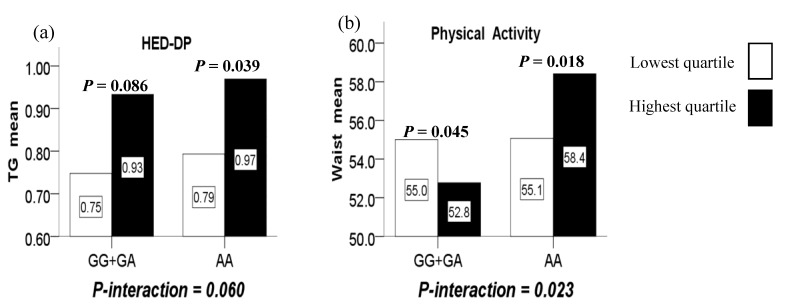
(**a**) The marginal interaction between the LMX1B rs10733682 polymorphism and HED-DP (high-energy density dietary patterns) on TG; (**b**) the interaction between the LMX1B rs10733682 polymorphism and physical activity duration on WC.

Furthermore, marginally significant interactions were observed between the LMX1B rs10733682 polymorphism and the HED-DP in terms of TG (*P*-interaction = 0.060). In such a way, compared with the lowest quartile, the highest quartile of the HED-DP increased TG level for the AA genotype (*P* = 0.039), while did not increase significantly for the GG+GA genotypes (*P* = 0.086). (Figure 2a) 

There was no significant association between the physical activity duration and obesity-related indicators, as well as genotypes (not shown). However, a significant interaction was observed between the LMX1B rs10733682 polymorphism and physical activity duration in terms of WC (*P*-interaction = 0.023). In such a way, the highest quartile of the physical activity duration compared to the lowest quartile, reduced WC level for girls with the GG+GA genotypes (*P* = 0.045) while increasing WC level for those AA genotype (*P* = 0.018). (Figure 2b).

## 4. Discussion

Due to gender, age, and regional differences, the boundaries of BMI and WHtR in different countries are not uniform. This study adopted the classification criteria proposed in the guidelines for the prevention and control of overweight and obesity in Chinese children and adolescents issued by the WGOC of the International Life Sciences Institute (ILSI) in 2004 [23]. Current domestic childhood obesity studies are basically based on criteria of this classification, thus the results in this study are comparable with others in China. It should be noted that, some scholars have proposed that BMI is a weaker predictor for relative body fat (%FAT) in children and adolescents [29]. Hence, further studies may consider using body fat percentage and total fat mass to assess obesity. A large number of studies have confirmed the use of anthropometry in predicting blood pressure, glucose, and lipid profile [30,31,32,33]. Results in our study also consistently demonstrated that overweight/obesity or central obesity children have significantly higher SP, DP, TG, LDL, and FBG, but significantly lower HDL levels compared to the normal weight. 

The intake of macronutrients is one aspect that affects the body’s energy balance. This study found that girls who consumed more total energy, fat, and carbohydrates, were associated with having higher TG level, but this level was inversely associated with the energy from protein. Surely, TG levels are also associated with high sugar and fructose intake, but they were contained in carbohydrates in this study, and need to be analyzed in more detail in the future. In addition, girls who had an increased energy from carbohydrate and more intake of dietary fiber were associated with having lower TC level. These results are in line with some previous studies for total energy [34], energy from protein [35], carbohydrates [36,37,38], and fiber intake [39,40], but inconsistent with fat [37,38]. Considering that only total fat intake was calculated in this study, further classification of saturated fat, monounsaturated fat, and polyunsaturated fat is needed in the future, since they have rather different effects on lipid profile and obesity.

The most attractive findings of this study were that the AA genotype of LMX1B rs10733682 was associated with greater incidence of overweight/obesity, central obesity, and higher body weight, WC, BMI, and WHtR; here showed for the first time in Han Chinese female children. In fact, all participants with the AA genotype also had a higher overweight/obesity prevalent rate than those with the GG+GA genotype, but such a difference in boys was not significant after gender stratification. For the sake of scientific rigor, we only further analyzed the situation of girls. In the future, we will confirm the relationship between the LMX1B rs10733682 polymorphism and obesity in boys by increasing the sample size. Several previous studies have discovered that the SNP of MC4R, FTO, leptin, and the respective receptor appear to be associated with higher energy and total lipid intake [13]. It’s worth noting that in this study, the interactions were also observed between the LMX1B rs10733682 SNP and intake of total energy, fat, as well as carbohydrate in terms of TG. The results showed that with the increased total energy, fat, and carbohydrate intake, the TG levels of girls with the AA genotype elevated, while those with GG+GA genotypes did not. This suggested that girls with the AA genotype are more genetically susceptible to elevation of TG levels by higher total energy, fat, and carbohydrate intake. Another interesting interaction was observed between the LMX1B rs10733682 polymorphism and energy from protein, that was, the highest quartile of energy intake from protein compared to the lowest quartile, displayed a significantly higher BMI and WC for the girls with the AA genotype, but a little lower for the GG+GA genotypes despite having no statistical difference. Previous studies showed that usual protein intake is inversely associated with BMI and WC in adults [41], and a higher protein intake in mid-childhood is associated with a higher fat-free mass [42]. Besides, the increased intake of carbohydrate reduced HDL in girls with the AA genotype while increasing for those GG+GA genotypes; and the increased fiber intake reduced the BMI in girls with the GG+GA genotypes while having no significant difference for those with AA genotype. Studies have shown a negative correlation between carbohydrate intake and HDL levels [43,44], and decreased HDL level is a well-known risk factor closely associated with obesity and metabolic syndrome [45].

On the other hand, the analysis of dietary patterns is considered a more realistic expression of dietary habits because it takes into account the complex interactions between nutrients and other components of the diet, making possible interventions to change eating habits [46,47]. In this study, four dietary patterns of girls were identified, of which HED-DP were associated with higher TG levels and VEF-DP was associated with lower levels of TC and LDL. The HED-DP similarly to western dietary pattern, mainly consists of pastries, meats and sweet foods, which is well-known more likely to cause hyperlipemia [48,49,50]. On the contrary, the VEF-DP mainly consists of vegetables, eggs and fishes, which are both nutritious and beneficial to lipids profile. Due to the fact that vegetables are fiber-rich [51] and fish are rich in n-3 PUFA [52], study has showed that TC and LDL can be reduced by changing the diet to plant-based and fish-based sources [53]. Moderate eggs intake can maintain LDL/HDL ratio and produces less LDL in children [54], so are worthwhile to be a part of a healthy eating pattern [55]. Furthermore, marginally interactions were observed that most inclining to HED-DP elevated the TG levels in girls with the AA genotype, and also in those with GG+GA genotypes despite not significant. This means that the AA genotype is more susceptible to the elevated TG caused by HED-DP.

To summarize, the AA genotype of LMX1B rs10733682 is a potential obesity and lipid profile risk factor in Han Chinese female children. This SNP can interact with related macronutrients intake, as well as dietary patterns more or less, but relevant molecular mechanisms were not involved in this study. The development and maintenance of obesity may involve central pathophysiological mechanisms such as brain circuit dysregulation and neuroendocrine hormone dysfunction [56]. Existing researches have demonstrated that when the LMX1B gene is functionally absent, the differentiation of serotonergic (5-HT) neurons is completely interrupted. It is an essential component of the mediation in the genetic cascade of early specialization and terminal differentiation of 5-HT neurons [57], and can therefore maintain the normal function of central 5-HT neurons [58]. Later, a study found that 5-HT neurons are likely to be the main regulator of energy balance throughout the body, playing a major role in regulating glucose and lipid homeostasis, and loss of 5-HT neurons can cause severe hyperglycemia and hyperlipidemia [59]. Therefore, the LMX1B gene can indirectly affect the regulation of energy balance in human body by affecting the development of 5-HT neurons. In fact, research into this molecular mechanism has only just begun, and our findings may provide clues for future research.

Emerging evidence supports a gene-physical activity interaction on obesity [60,61,62]. In our study, the LMX1B rs10733682 SNP significant interacted with physical activity duration in term of WC. Consistent with the results of the GG+GA genotype group, studies have revealed that physical activity level is negatively correlated with the WC [63,64]. However, the positive correlation between the WC and physical activity duration was found in the girls with AA genotype in our study, in other words, more physical activity did not decrease but increase the WC. It seems a somewhat inexplicable result, to be confirmed by future large sample investigation.

Finally, due to the cross-sectional design, causality cannot be inferred, and more in-depth cellular and molecular biological experimental studies are needed to discover the precise mechanism. Although factor analysis was used to define dietary pattern, it is somewhat subjective. Besides, the interaction between SNPs is another aspect that needs to be studied in the future. Anyway, based on PubMed search, this is the first study to preliminarily demonstrate the relationship between the LMX1B rs10733682 SNP and childhood obesity, macronutrients intake and dietary patterns, as well as physical activity.

## 5. Conclusions

This study is the first to indicate the relationship between a novel SNP of LMX1B rs10733682 and general overweightness, obesity, and central obesity in Han Chinese girls. The AA genotype was probably a potential risk factor for obesity and the lipid profile. Subsequently, it was shown that the AA genotype can interact with an increased intake of total energy, fat, and carbohydrates, causing an increase of TG, and can also interact with increased energy from proteins, causing an increase of BMI and WC. Furthermore, girls inclining to the HED-DP were associated with higher TG levels, but those inclining to the VEF-DP were associated with lower TC and LDL levels. Girls with the AA genotype were also more susceptible to elevated TG caused by the HED-DP than those with the GG+GA genotypes. All of these results provide valuable clues for the individualized prevention and intervention of childhood obesity in China.

## Figures and Tables

**Table 1:** Obesity-related indicators according to the LMX1B rs10733682 genotypes of girls.

Variables	GG+GA (*n* = 157)	AA (*n* = 241)	*P* ^1^	*P*-Ancova ^2^
Weight (kg)	29.5 ± 6.2	31.0 ± 7.8	0.035	0.012
BMI	16.2 ± 2.2	16.7 ± 2.7	0.049	0.040
WC (cm)	54.29 ± 6.01	56.02 ± 7.55	0.016	0.010
WHtR	0.40 ± 0.04	0.41 ± 0.05	0.044	0.046
BMR	1137.4 ± 115.8	1164.9 ± 139.3	0.040	0.029
SP (mmHg)	99 ± 10	99 ± 10	0.738	0.721
DP (mmHg)	61 ± 6	62 ± 6	0.548	0.541
TG (mM)	0.80 ± 0.42	0.88 ± 0.48	0.079	0.091
TC (mM)	3.89 ± 0.82	3.87 ± 0.87	0.808	0.823
HDL (mM)	1.39 ± 0.28	1.41 ± 0.25	0.574	0.563
LDL (mM)	2.32 ± 0.60	2.30 ± 0.69	0.757	0.729
FBG (mM)	4.66 ± 0.40	4.63 ± 0.37	0.332	0.324

Note: variables are expressed as mean value ± SD; (n) sample size. ^1^ For the crude ANOVA model. ^2^ For the adjusted ANCOVA model by age, total energy intake, and physical activity duration. Abbreviations: BMI (body mass index), WC (waist circumference), WHtR (waist-to-height ratio), BMR (basal metabolic rate); SP (systolic pressure), DP (diastolic pressure), TG (triglyceride), TC (total cholesterol), HDL (high density lipoprotein), LDL (low density lipoprotein), FBG (fasting blood glucose).

**Table 2:** Obesity-related indicators compared by general overweight/obesity, central obesity and normal weight of girls.

Variables	Normal Weight (*n* = 334)	Overweight/Obesity (*n* = 64)	*P*-Value ^1^	Normal Weight (*n* = 345)	Central Obesity (*n* = 53)	*P*-Value ^1^
SP (mmHg)	98 ± 9	104 ± 9	<0.001	98 ± 9	104 ± 10	<0.001
DP (mmHg)	61 ± 6	64 ± 6	<0.001	61 ± 6	64 ± 6	0.001
TG (mM)	0.83 ± 0.46	0.96 ± 0.44	0.035	0.82 ± 0.45	1.01 ± 0.49	0.005
TC (mM)	3.84 ± 0.87	4.03 ± 0.70	0.059	3.87 ± 0.83	3.93 ± 0.94	0.616
HDL (mM)	1.42 ± 0.26	1.32 ± 0.25	0.004	1.42 ± 0.26	1.29 ± 0.26	0.001
LDL (mM)	2.27 ± 0.66	2.51 ± 0.62	0.006	2.27 ± 0.66	2.51 ± 0.61	0.013
FBG (mM)	4.62 ± 0.38	4.74 ± 0.38	0.026	4.63 ± 0.39	4.71 ± 0.35	0.121

Note: Variables are expressed as mean value ± SD; (n) sample size. ^1^ For independent t-test. Overweight/obesity was defined as a BMI greater than 17.2 (7 years old ~), 18.1 (8 years old ~), 19.0 (9 years old~), 20.0 (10 years old ~) [23], and central obesity was defined as a WHtR greater than 0.45 [24]. Abbreviations: SP (systolic pressure), DP (diastolic pressure), TG (triglyceride), TC (total cholesterol), HDL (high density lipoprotein), LDL (low density lipoprotein), FBG (fasting blood glucose).

**Table 3:** Obesity-related indicators compared by quartile of macronutrients intake of girls.

	Quartile 1	Quartile 2	Quartile 3	Quartile 4	*P* ^1^	*P*-Ancova ^2^
**TG (mM)**						
Total energy intake	0.80 ± 0.46	0.76 ± 0.36	0.87 ± 0.45	0.98 ± 0.53	0.006	0.007
Energy from protein	0.94 ± 0.48	0.86 ± 0.52	0.80 ± 0.39	0.79 ± 0.40	0.022	0.037
Energy from fat	0.73 ± 0.36	0.88 ± 0.45	0.91 ± 0.48	0.86 ± 0.51	0.052	0.067
Energy from carbohydrate	0.86 ± 0.52	0.85 ± 0.43	0.82 ± 0.40	0.87 ± 0.49	0.848	0.847
Protein intake	0.83 ± 0.47	0.80 ± 0.43	0.84 ± 0.45	0.93 ± 0.49	0.291	0.292
Fat intake	0.77 ± 0.36	0.78 ± 0.40	0.88 ± 0.51	0.97 ± 0.52	0.005	0.005
Carbohydrate intake	0.76 ± 0.44	0.83 ± 0.39	0.81 ± 0.42	1.00 ± 0.54	0.002	0.002
Dietary fiber intake	0.81 ± 0.49	0.81 ± 0.40	0.88 ± 0.46	0.89 ± 0.49	0.457	0.458
**TC (mM)**						
Total energy intake	4.02 ± 0.87	3.90 ± 0.80	3.76 ± 0.77	3.82 ± 0.94	0.149	0.184
Energy from protein	3.73 ± 0.83	3.89 ± 0.86	3.95 ± 0.83	3.95 ± 0.86	0.210	0.151
Energy from fat	3.81 ± 0.74	3.90 ± 0.99	3.87 ± 0.86	3.91 ± 0.76	0.850	0.863
Energy from carbohydrate	4.03 ± 0.81	3.92 ± 0.94	3.88 ± 0.76	3.65 ± 0.83	0.017	0.015
Protein intake	3.89 ± 0.91	3.95 ± 0.77	3.87 ± 0.80	3.79 ± 0.89	0.647	0.633
Fat intake	3.92 ± 0.86	3.92 ± 0.78	3.88 ± 0.88	3.76 ± 0.86	0.500	0.550
Carbohydrate intake	4.02 ± 0.82	3.94 ± 0.83	3.78 ± 0.91	3.74 ± 0.80	0.022	0.039
Dietary fiber intake	4.04 ± 0.87	3.92 ± 0.95	3.78 ± 0.81	3.78 ± 0.73	0.029	0.035

Note: variables are expressed as mean value ± SD. ^1^ For the crude ANOVA model. ^2^ For the adjusted ANCOVA model by age and physical activity duration. Abbreviations: TG (triglyceride), TC (total cholesterol). The mean values of energy and macronutrients intake for each quartile are as follow: Total energy intake (kcal/d): Q1(787 ± 157), Q2(1172 ± 94), Q3(1502 ± 102), Q4(2529 ± 221); Energy from protein (%): Q1(15.5 ± 2.4), Q2(19.7 ± 1.0), Q3(23.1 ± 1.0), Q4(28.4 ± 3.1); Energy from fat (%): Q1(19.2 ± 3.9), Q2(26.9 ± 1.6), Q3(32.1 ± 1.7), Q4(41.7 ± 4.8); Energy from carbohydrate (%): Q1(40.2 ± 4.5), Q2(50.1 ± 1.9), Q3(56.5 ± 1.8), Q4(66.7 ± 5.6); Protein intake (g/d): Q1(25.9 ± 6.2), Q2(40.3 ± 3.9), Q3(54.1 ± 4.4), Q4(88.9 ± 8.4); Fat intake (g/d): Q1(14.3 ± 4.0), Q2(24.2 ± 2.5), Q3(34.5 ± 3.5), Q4(68.4 ± 4.5); Carbohydrate intake (g/d): Q1(69.5 ± 14.1), Q2(106.5 ± 9.5), Q3(139.9 ± 12.2), Q4(232.1 ± 10.0); Dietary fiber intake (g/d): Q1(1.9 ± 0.6), Q2(3.6 ± 0.5), Q3(5.5 ± 0.6), Q4(12.8 ± 0.4).

**Table 4:** Factor loadings for the four identified dietary patterns of girls.

Food Groups	Dietary Patterns			
	HED-DP	NPW-DP	VEF-DP	DFE-DP
Pastries	0.749			
Other meats	0.736			
Poultry	0.410			
Sweet foods	0.400			0.361
Dairy				0.722
Fruits				0.717
Vegetables			0.657	
Eggs			0.431	0.411
Fish			0.373	
Nuts		0.767		
Pork		0.692		
Wheat	0.373	0.569		
Eigenvalues	1.901	1.716	1.666	1.512
Variance (%)	9.1	8.2	7.9	7.2
Total variance (%) = 32.4				

Note: values are factor loadings of dietary patterns measured by factor analysis. Factor loadings below ±0.35 are not shown. Abbreviations: HED-DP (high-energy density dietary patterns); NPW-DP (nuts, pork, and wheat-based dietary patters); VEF-DP (vegetables, eggs, and fishes based dietary patterns); DFE-DP (dairy, fruits, eggs-based dietary patterns).

**Table 5:** Characteristics by quartile (Q) of dietary patterns for girls.

Characteristics	HED-DP		*P*	NPW-DP		*P*	VEF-DP		*P*	DFE-DP		*P*
	Q1 (n = 100)	Q4 (n = 100)		Q1 (n = 100)	Q4 (n = 99)		Q1 (n = 100)	Q4 (n = 99)		Q1 (n = 100)	Q4 (n = 99)	
Ag (year)	9.0 ± 0.9	9.2 ± 0.9	0.066	9.1 ± 0.9	9.3 ± 0.8	0.059	9.2 ± 0.9	9.2 ± 1.0	0.594	9.2 ± 0.9	9.0 ± 0.9	0.264
Overweight & obesity (%)	17.0	12.0	0.329	17.0	14.1	0.360	12.0	17.2	0.322	21.0	16.2	0.467
Central obesity (%)	10.0	13.0	0.211	12.0	12.1	0.576	10.0	16.2	0.214	18.0	12.1	0.322
TG	0.78 ± 0.37	0.95 ± 0.54	0.008	0.90 ± 0.51	0.90 ± 0.48	0.917	0.77 ± 0.37	0.91 ± 0.51	0.132	0.84 ± 0.46	0.91 ± 0.47	0.252
TCH	3.87 ± 0.75	3.83 ± 0.94	0.753	3.78 ± 0.87	3.82 ± 0.82	0.731	4.06 ± 0.84	3.70 ± 0.93	0.045	3.87 ± 0.84	3.82 ± 0.95	0.670
LDL	2.25 ± 0.62	2.39 ± 0.74	0.153	2.25 ± 0.59	2.31 ± 0.67	0.511	2.46 ± 0.66	2.17 ± 0.57	0.016	2.22 ± 0.58	2.39 ± 0.72	0.072
Genotype (%)			0.424			0.512			0.210			0.451
GG+GA	21.7	25.5		28.7	25.5		23.6	22.9		23.6	28.0	
AA	27.4	24.9		22.8	24.5		26.1	26.1		26.1	22.8	
BER (kcal/d)	1149 ± 141	1149 ± 122	0.991	1159 ± 152	1151 ± 108	0.665	1145 ± 116	1149 ± 128	0.809	1168 ± 139	1157 ± 145	0.565
Physical activity (hour/d)	2.6 ± 1.5	2.6 ± 1.4	0.982	2.5 ± 1.3	2.7 ± 1.3	0.265	2.8 ± 1.4	2.7 ± 1.4	0.885	2.7 ± 1.4	2.5 ± 1.3	0.416
Total energy intake (kcal/d)	1440 ± 433	1861 ± 522	0.021	1529 ± 463	1736 ± 593	0.376	1570 ± 472	1632 ± 576	0.652	1582 ± 443	1778 ± 579	0.367
En% of protein	21.2 ± 4.8	19.0 ± 5.8	0.003	20.0 ± 5.2	20.5 ± 5.5	0.444	19.4 ± 5.1	20.8 ± 4.9	0.291	21.2 ± 6.3	20.2 ± 3.8	0.101
En% of fat	29.3 ± 9.2	28.9 ± 9.0	0.752	26.8 ± 8.7	29.7 ± 9.4	0.026	25.0 ± 8.9	29.8 ± 7.7	0.011	27.4 ± 8.9	28.7 ± 8.1	0.161
En% of carbohydrate	49.5 ± 10.5	52.1 ± 10.3	0.042	53.2 ± 10.6	49.8 ± 10.7	0.034	55.6 ± 10.2	49.4 ± 9.3	0.001	51.4 ± 11.3	51.1 ± 9.0	0.643

Categorical variables are presented as percentages, and continuous variables are presented as mean ± standard deviation (SD). *P* values for continuous variables (analysis of variance) and for categorical variables (chi-square test). Quartiles of dietary pattern are presented by Q1 (lowest), Q4 (highest). Abbreviations: HED-DP (high-energy density dietary patterns); NPW-DP (nuts, pork, and wheat-based dietary patters); VEF-DP (vegetables, eggs, and fishes based dietary patterns); DFE-DP (dairy, fruits, eggs-based dietary patterns); BER (basal metabolic rate); En%: percent of energy intake.
